# Trimetazidine therapy for diabetic mouse hearts subjected to *ex vivo* acute heart failure

**DOI:** 10.1371/journal.pone.0179509

**Published:** 2017-06-20

**Authors:** Emilene Breedt, Lydia Lacerda, M. Faadiel Essop

**Affiliations:** Cardio-Metabolic Research Group (CMRG), Department of Physiological Sciences, Stellenbosch University, Stellenbosch, South Africa; Brown University, UNITED STATES

## Abstract

Acute heart failure (AHF) is the most common primary diagnosis for hospitalized heart diseases in Africa. As increased fatty acid β-oxidation (FAO) during heart failure triggers detrimental effects on the myocardium, we hypothesized that trimetazidine (TMZ) (partial FAO inhibitor) offers cardioprotection under normal and obese-related diabetic conditions. Hearts were isolated from 12-14-week-old obese male and female diabetic (*db/db*) mice versus lean non-diabetic littermates (*db/+*) controls. The Langendorff retrograde isolated heart perfusion system was employed to establish an *ex vivo* AHF model: a) Stabilization phase—Krebs Henseleit buffer (10 mM glucose) at 100 mmHg (25 min); b) Critical Acute Heart Failure (CAHF) phase–(1.2 mM palmitic acid, 2.5 mM glucose) at 20 mmHg (25 min); and c) Recovery Acute Heart Failure phase (RAHF)–(1.2 mM palmitic acid, 10 mM glucose) at 100 mmHg (25 min). Treated groups received 5 μM TMZ in the perfusate during either the CAHF or RAHF stage for the full duration of each respective phase. Both lean and obese males benefited from TMZ treatment administered during the RAHF phase. Sex differences were observed only in lean groups where the phases of the estrous cycle influenced therapy; only the lean follicular female group responded to TMZ treatment during the CAHF phase. Lean luteal females rather displayed an inherent cardioprotection (without treatments) that was lost with obesity. However, TMZ treatment initiated during RAHF was beneficial for obese luteal females. TMZ treatment triggered significant recovery for male and obese female hearts when administered during RAHF. There were no differences between lean and obese male hearts, while lean females displayed a functional recovery advantage over lean males. Thus TMZ emerges as a worthy therapeutic target to consider for AHF treatment in normal and obese-diabetic individuals (for both sexes), but only when administered during the recovery phase and not during the very acute stages.

## Introduction

Cardiovascular diseases (CVD) remain the leading cause of global mortality accounting for ~30% of all deaths worldwide [1, 2]. Future projections also indicate that both developed and developing nations face a growing CVD burden, e.g. heart failure prevalence is expected to increase by 25% by 2030 [1]. Moreover, the higher incidence of obesity and diabetes—due to poor lifestyle choices and inadequate control of CVD risk factors—will further fuel this growing burden of disease [2].

Acute heart failure (AHF) is a complex clinical syndrome that varies extensively in terms of underlying pathophysiologic mechanisms and clinical presentations [3]. AHF results in a 3–8% in-hospital mortality rate, a 9–13% 60–90 day mortality rate and a 25–30% short term re-hospitalization rate; and as re-hospitalizations within one year can reach 50%, this demonstrates the severity of this disease [4]. Acute heart failure syndromes (AHFS) are most commonly diagnosed in US Medicare patients, resulting in hospitalization and significant expenses [5, 6]. By contrast, acute decompensated heart failure (ADHF) is the most common primary diagnosis in patients with heart disease admitted to hospitals in Africa [7, 8]. While AHF is usually a disease of the elderly (mean age 70–72 years) in developed nations [9, 10], it strikes at a mean age of around 52 years in African countries [11, 12]. Although registries of higher income countries reveal that women with AHFS are generally older than men [13,14], sub-Saharan Africa women are on average younger and more prone to *de novo* AHF compared to men [11].

Co-morbidities may act as a trigger for AHF and also play a contributory role in pathology onset and progression [15]. For example, a graded pattern manifests when AHF patients are divided into body mass index (BMI) quartiles, i.e. as BMI increases patients are younger and display more diabetes. In addition, for the fourth quartile (highest BMIs) more AHF patients tend to be female [16]. Of note, perturbations of metabolic substrate utilization are strongly linked to both obesity (with concomitant Type 2 diabetes) and heart failure onset and progression. For example, increased fatty acid β-oxidation (FAO) together with a concomitant decrease in glucose oxidation (GO) can elicit detrimental effects within the myocardium by attenuating cardiac function and potentially resulting in death [17, 18].

As for any acute onset event, diagnosis and treatments for AHFS are generally performed in parallel fashion. During this process the close monitoring of patients is crucial as any change(s) in condition may require rapid implementation of a different therapeutic regimen(s). For further treatment of suspected/confirmed AHFS the immediate goal is to improve symptoms and to stabilize the hemodynamic condition [19,20]. However, the generic nature of such interventions shows that current therapies are not specifically designed for AHF. Alternative treatment modalities were recently proposed but were generally unsuccessful as it did not lower re-hospitalization rates or improve patient outcome [21]. Thus an important issue to be addressed is the relatively slow progress to develop AHF-specific therapies [22, 23].

In light of the apparent lack of success with hemodynamically-related drug regimens, the current pre-clinical study set out to evaluate whether cardiac metabolic modulation can be employed as a novel therapeutic approach for AHF treatment. Although studies investigating the use of metabolic moderators have yielded inconsistent data, Trimetazidine (TMZ) emerges as a robust therapeutic option as it is a commonly used metabolic modulator that is available in more than 80 countries [24]. This anti-anginal agent acts by selectively inhibiting long-chain 3-ketoacyl CoA thiolase (3-KAT), thereby attenuating cardiac FAO and shifting metabolism to GO via the Randle cycle [25, 26]. Although beneficial effects of TMZ were reported for unspecified, ischemic and non-ischemic HF [27, 28], it remains unclear whether it can enhance cardiac function within the context of AHF. Due to the robust incidence of obesity and diabetes (generally associated with higher FAO rates) in individuals suffering from AHF, we set out to assess TMZ-mediated cardioprotective effects in this sub-group versus lean counterparts subjected to AHF.

This study also examined sex-based differences as the CVD risk of females were undervalued in the past due to the misunderstanding that only estrogen is responsible for cardioprotection in this group [29]. However, this misconception has since been addressed [30,31] and multiple campaigns have since promoted the inclusion of female subjects in pre-clinical and clinical studies. Here the focus is largely on the effects of estrogen and (to a lesser extent) progesterone on the heart [32–35] and also its impact on energy metabolism. For example, estrogen can decrease muscle GO, gluconeogenesis and glycogenolysis while elevating FAO [36]. By contrast, the known effects of progesterone on the heart are less well understood. However, some found that physiologically high progesterone levels are associated with increased congestive heart failure prevalence [37], while the first clues of its role in energy metabolism only emerged more recently, i.e. mediating its effects via the progesterone receptor-α to subsequently activate PI3-K/AKT-1 and MAPK in addition to increased endothelial nitric oxide (eNOS) activity and NO production [38, 39].

In light of this, we hypothesized that TMZ acts as a novel cardioprotective agent during AHF under normal and obese-related diabetic conditions. It is further hypothesized that this response will vary depending on sex and that the estrous cycle will play a key role in this instance. To better assess the latter notion we further divided females into the two main phases of the estrous cycle, i.e. the follicular phase (estrogen predominates) and the luteal phase (progesterone predominates). The main aims of this study were: (1) to establish a *de novo*, *ex vivo* AHF mouse model; and (2) to evaluate the therapeutic value of TMZ under normal and obese-related diabetic conditions in both male and female mouse hearts subjected to *ex vivo* AHF. We also evaluated the influence of estrous cycle phases (follicular, luteal) on TMZ efficacy to gain additional insights regarding the nature of potential sex differences.

## Methods

### Animals and ethics

Animals were treated in agreement with the Guide for the Care and Use of Laboratory Animals of the National Academy of Science (National Institutes of Health publication No. 85–23, revised 1996). This study was executed with the approval of the Animal Ethics Committees of Stellenbosch University (South Africa). For the experimental procedure, 102 male and female diabetic (*db/db*) mice (aged 12–14 weeks) and their lean non-diabetic littermates (*db/+*) were used; original breeding pairs were purchased from Jackson Laboratories (Bar Harbor, ME). Mice were housed in the animal housing facility (n = 6 per cage) at Stellenbosch University, under standard conditions and had *ad libitum* access to water and standard chow.

### Vaginal cytology

The estrous cycle of females (12–14 weeks old) was determined by vaginal smear cytology or “wet smears” as previously described [40, 41]. As group-housed female mice do not display reliable regular estrous cycling [42, 43], bedding from young male mice was placed in female cages for a minimum of 5 days prior to performing estrous phase determination. Vaginal secretion was collected by inserting a pipette tip (filled with 10 μl saline) into the vaginal opening, taking care not to insert it too deep in order to prevent harming the female. The saline was injected and the vaginal flush was collected in the same tip which was then transferred to a clean glass slide (Labstar frosted microscope slides; Lasec Laboratory Solution Provider, Cape Town South Africa). The unstained material was viewed under a light microscope (YS 100, Nikon Co., Chiyoda, Tokyo Japan) (without use of condenser lens) under 4x, 10x and 40x objective lenses. Three distinct cell types were present, i.e. epithelial cells (round and nucleated), cornified epithelial cells (irregular in shape, absence of a nucleus) and leukocytes (black, small and round). The ratio of cell types in the vaginal flush was used to determine estrous cycle phases, i.e. proestrus was characterized by the predominance of epithelial cells; estrus by the majority of cornified epithelial cells; metestrus by the presence of a mixed cell type dominated by leukocytes and diestrus by the bulk of leukocytes.

### Acute heart failure: Retrograde Langendorff heart perfusion

Male and female mice (immediately following estrous phase determination) were anesthetized by an intramuscular injection of ketamine (100 mg/kg) and xylazine (5 mg/kg). Thereafter hearts of the 12–14 week old mice were rapidly excised into ice-cold perfusion buffer and mounted on a cannula connected to the modified Langendorff retrograde perfusion model—flow from the stabilization phase was immediately commenced. The left atrial appendage was cut off and a deflated self-made balloon constructed from cling wrap (connected to a distilled water-filled catheter [Portex Jackson catheter with luer connection (4F), Smiths Medical International Ltd., Kent UK]) was placed into the left ventricle via the mitral valve. The balloon was inflated using an insulin syringe connected to the pressure transducer (Stratham MLT 0380/D, AD Instruments Inc., Bella Vista, NSW Australia) compatible with the PowerLab System ML410/W (AD Instruments Inc., Bella Vista, NSW Australia). A pacer was connected to the right atrial appendage of the heart and set to 400 bpm. A buffer-filled reservoir—correlating to each phase—was raised around the excised heart to maintain the heart at a stable temperature of 37.5°C. The PowerLab System was set to record the following functional parameters: left ventricular end diastolic pressure (LVEDP), left ventricular end systolic pressure (LVESP), left ventricular developed pressure (LVDevP) [calculated as LVESP-LVEDP], heart rate, rate pressure product (RPP) calculated as [heart rate x LVDevP] and (dp/dt)_max_ (maximum velocity of contraction) [calculated as pressure difference in mmH_2_O/time in sec]. Data were recorded for each parameter listed and for every animal included in this study. Thereafter the data (for each animal) were expressed as either a percentage of the baseline or as a percentage of the previous phase. After this step, the final data presented were derived by averaging such values for each experimental group.

The mouse *ex vivo* Langendorff retrograde perfusion model of *de novo* AHF was modified from a previously established rat model [44–46]. Each of the three phases (discussed below) was maintained for 25 minutes, for total protocol duration of 75 minutes. All buffers were filtered through a low protein binding filter (Durapore Polyvinylidene fluoride [PVDF] 0.22 μm, Merck Millipore Co., Darmstadt Germany) on the day of perfusion. The model consists of three phases namely (1) stabilization, (2) critical acute heart failure (CAHF), and (3) recovery acute heart failure (RAHF). The buffer for all three phases was based on mouse Krebs-Henseleit buffer (NaCl 118 mM, NaHCO_3_ 24 mM, KCl 4.02 mM, NaH_2_PO_4_ 1.16 mM, Disodium EDTA.2H_2_0 0.5 mM, MgCl_2_.6H_2_0 2.5 mM, CaCl_2_ 3.3 mM), with deviations only in glucose and FA concentrations. The stabilization phase was perfused at 100 cmH_2_O perfusion pressure with glucose (10 mM/L) as the sole substrate. Hearts that did not achieve a stable heart rate of 400 ± 20 beats per minute (bpm) and a left ventricular developed pressure of >20 mmHg were excluded from the study. The CAHF phase is perfused at a reduced perfusion pressure of 20 cmH_2_O and here the glucose concentration is decreased to 2.5 mM/L, while introducing 1.2 mM/L FA (palmitic acid conjugated to 3% bovine serum albumin [BSA]) into the buffer. This phase was allowed to continue without any pacing. For the RAHF phase the perfusion pressure was restored back to 100 cmH_2_O with the pacer turned on again. Glucose concentrations were also restored to 10 mM/L baseline condition, with the high FA content (1.2 mM/L) being maintained in the recovery buffer. All buffers were gently gassed with 95% O_2_/5% CO_2_ (Afrox, Gauteng South Africa) for 20 minutes prior to perfusion and for the duration of the experiment.

#### TMZ treatment

Control groups were allowed to go through the full protocol without any interventions. However, treated groups received TMZ (5 μM) (Sigma-Aldrich, St. Louis MO) in the perfusate in either the CAHF or RAHF stage for the full duration of each respective phase. The therapeutic dose was selected based on mean plasma TMZ concentrations in a clinical study [47] and translated to an equivalent animal dosage as previously described [48].

### Statistical analysis

Data are expressed as mean ± standard error of the mean (SEM) and were analyzed using either a one-way or two-way analysis of variance (ANOVA) where appropriate, followed by a Bonferroni *post-hoc* test (GraphPad Prism v5, San Diego CA). A value of p ≤ 0.05 was considered significant.

## Results

### Characterizing the estrous cycle

**Fig 1:**
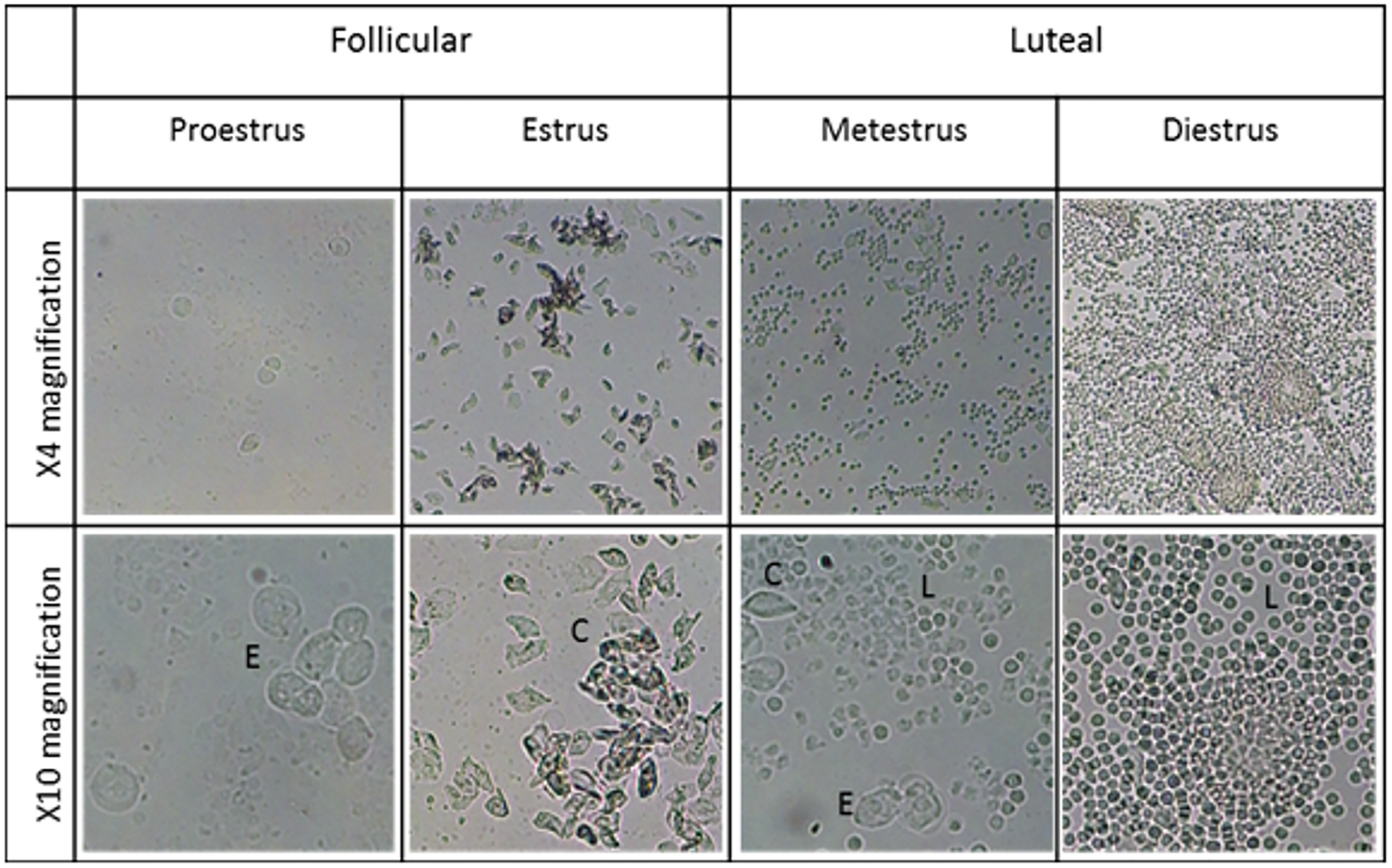
Stages of the estrous cycle characterized by vaginal cytology. The phases are characterized by the ratio of cells that are present. Indicated are nucleated epithelial cells (E), cornified squamous epithelial cells (C) and leucocytes (L). Images represent the vaginal flush of lean females under 4x and 10x magnification.

The different stages of the estrous cycle were initially identified, i.e. the follicular (proestrus and estrus) and luteal (metestrus and diestrus) ones. Slides prepared from mice in the proestrus phase showed a predominance of nucleated epithelial cells and occasionally some cornified cells and/or leukocytes were present (Fig 1). During the estrus phase there was a predominance of cornified squamous epithelial cells that are easily identifiable by its irregular shape and granular cytoplasm. The metestrus phase was characterized by the presence of mixed cell types although a predominance of leukocytes occurs, while the diestrus phase is completely dominated by exceptionally dense depositions of leukocytes (Fig 1). While all four phases were detected in lean females, obese females did not undergo any follicular phases.

### Establishing the *ex vivo* AHF mouse model

**Fig 2:**
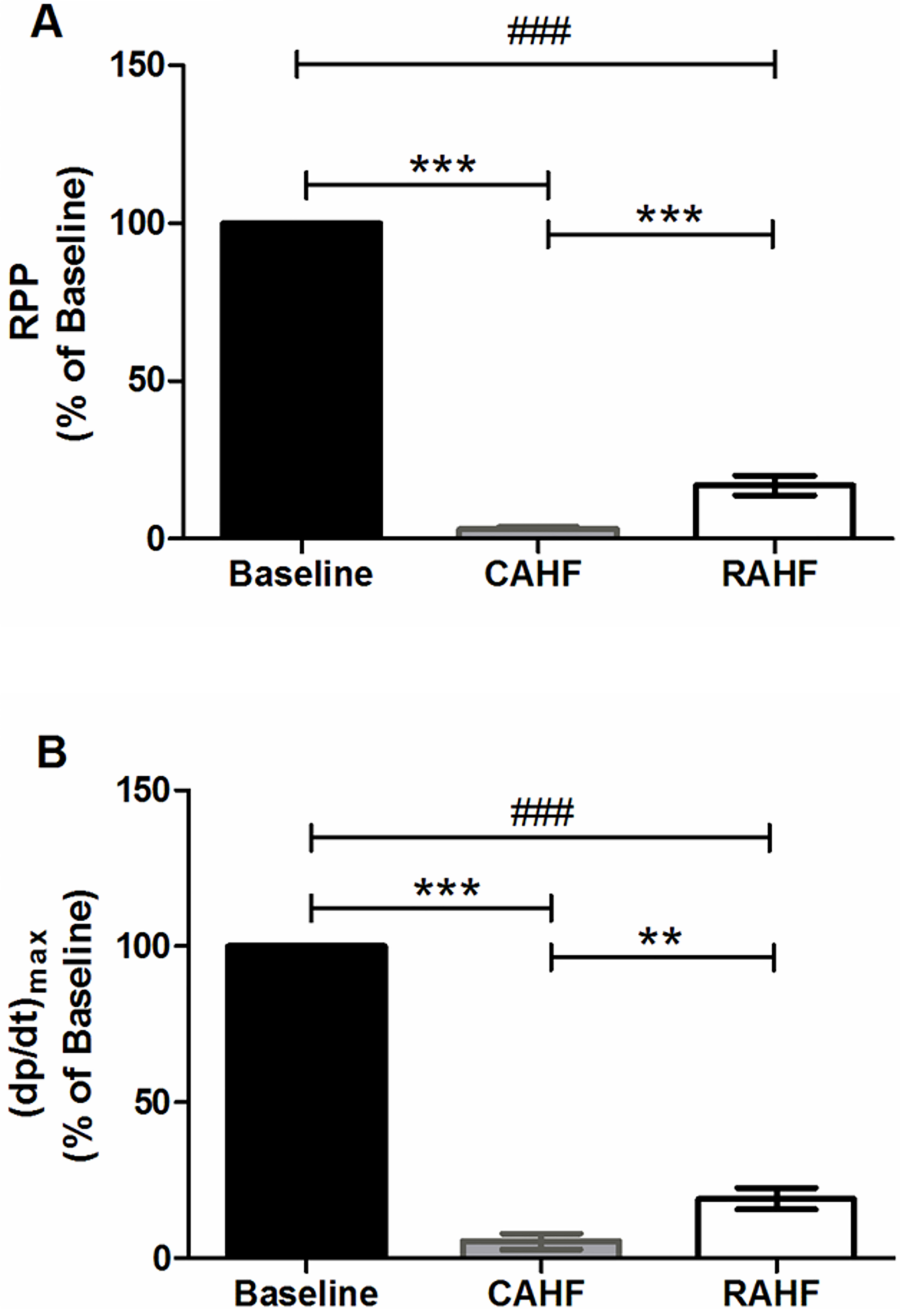
RPP and (dp/dt)_max_ in establishing the ex vivo mouse model of acute heart failure (AHF) in lean male mice. Data is expressed as a percentage of baseline (n = 6). Data represent the last ten minutes of each phase. Significance is expressed as **p<0.01; ***p<0.001 versus CAHF; and ###p<0.001 versus RAHF. RPP: rate pressure product, (dp/dt)_max_: index of myocardial contraction velocity, CAHF: critical acute heart failure, RAHF: recovery acute heart failure.

The rapid switch in the perfusion system from the stabilization to the CAHF phase (reflecting *de novo* AHF) resulted in a drastic decrease in both RPP (p<0.001) and (dp/dt)_max_ (p<0.001) versus controls (Fig 2A and 2B). Switching from the CAHF to the RAHF phase (reflecting partial recovery) resulted in modest increases for RPP (p<0.001) and (dp/dt)_max_ (p<0.01) (Fig 2A and 2B, Table 1). However, the resultant recovery in the RAHF phase was still statistically lower than the initial baseline for both RPP (p<0.001) and (dp/dt)_max_ (p<0.001). There were no significant changes in heart rate between the phases, although there was a definite tendency for higher heart rates in the CAHF phase compared to the baseline. Heart rate during the RAHF phase was also slightly elevated compared to baseline (not significant), indicating that the heart sustained damage even though there is some recovery (Table 1). LVEDP decreased following the switch from baseline to CAHF (p<0.01) and subsequently increased during the RAHF phase compared to CAHF (p<0.001). There was a tendency for the LVEDP to be higher during RAHF compared to baseline, although this was not statistically significant. The LVESP followed a similar pattern, although it was lower during RAHF versus the initial baseline (p<0.05) (Table 1).

**Table 1:** Establishing the *ex vivo* mouse heart model of AHF (lean male mice).

Parameters	Baseline vs. CAHF	CAHF Phase (% of Baseline)	CAHF vs. RAHF	RAHF Phase (% of Baseline)	Baseline vs. RAHF
Heart rate (%)	ns	274.70 ± 81.42	ns	118.90 ± 12.72	ns
LVDevP (%)	***	6.40 ± 4.12	ns	15.23 ± 3.31	###
LVEDP (%)	**	65.06 ± 6.89	***	120.30 ± 6.60	ns
LVESP (%)	***	46.47 ± 5.75	***	86.11 ± 1.61	#

Data from the CAHF and RAHF phases were normalized to baseline and thus the CAHF and RAHF phases data are expressed as a percentage of baseline (n = 6). Data represent the last ten minutes of each phase. Significance is expressed as **p<0.01; ***p<0.001 vs. CAHF and #p<0.05; ###p<0.001 vs. RAHF. LVDevP: left ventricular developed pressure; LVEDP: left ventricular end diastolic pressure; LVESP: left ventricular end systolic pressure; CAHF: critical acute heart failure; RAHF: recovery acute heart failure; ns: not significant.

### TMZ treatment of male hearts subjected to AHF

**Fig 3:**
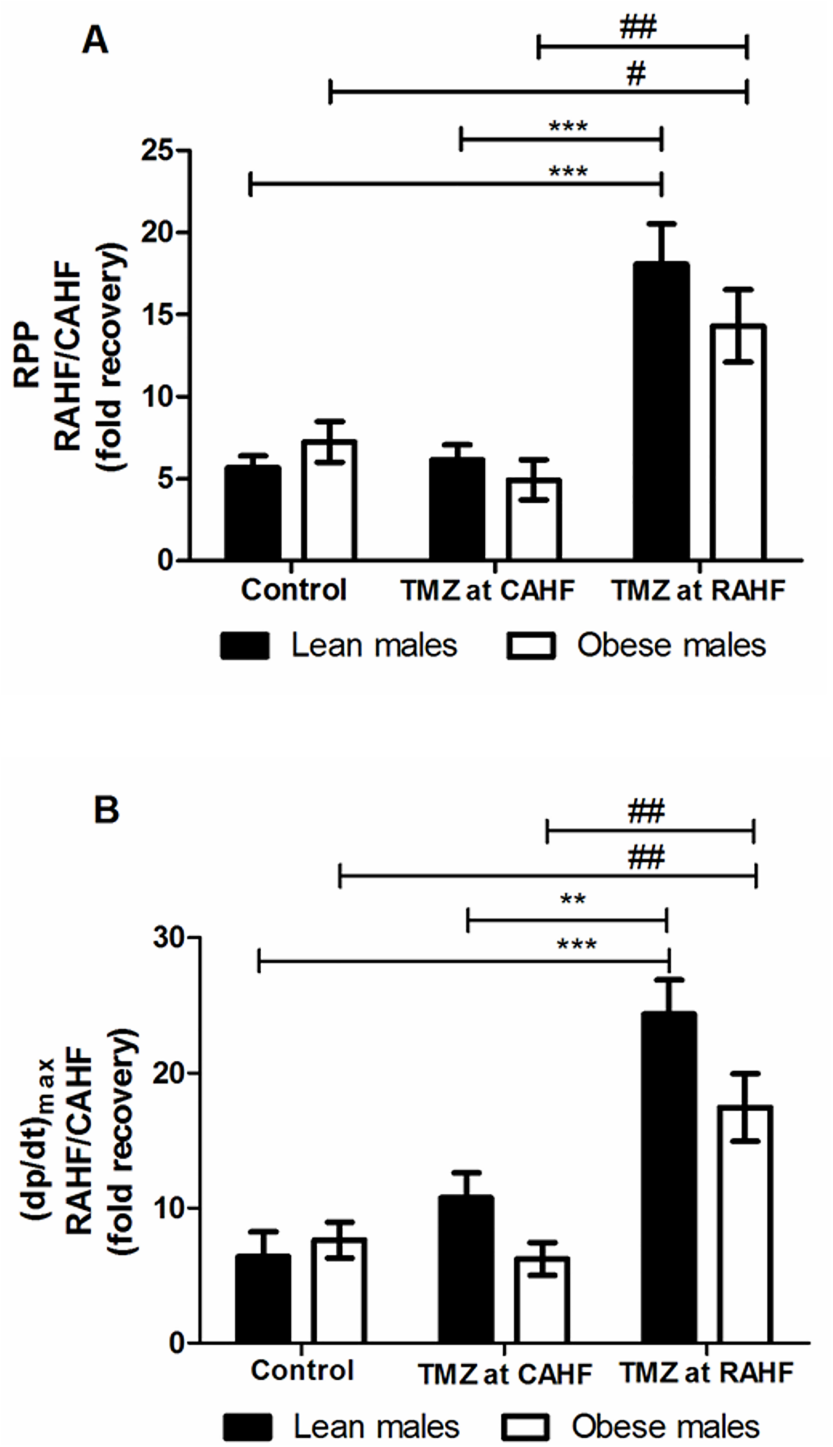
Effect of TMZ treatment on RPP and (dp/dt)_max_ of lean and obese males. (A) RPP (B) (dp/dt)max (n = 6). Data represent the last ten minutes of the RAHF phase and expressed as a fold recovery from the previous CAHF phase. Significance is expressed as **p<0.01; ***p<0.001 vs. TMZ at RAHF (lean males); and #p<0.05; ##p<0.01 vs. TMZ at RAHF (obese males). RPP: rate pressure product; (dp/dt)_max_; index of myocardial contraction velocity; CAHF: critical acute heart failure; RAHF: recovery acute heart failure.

The control lean males showed a 5.6- and 6.4-fold increase for RPP and (dp/dt)_max_, respectively, from the previous CAHF phase. TMZ treatment initiated during the CAHF phase did not lead to any differences in terms of functionality. However, when TMZ treatment was initiated during the RAHF phase this strongly improved RPP and (dp/dt)_max_ values compared to both control and CAHF treated lean males (Fig 3A and 3B, Table 2). Here data revealed an impressive 3.2-fold increase in RPP between RAHF-treated and control lean males (p<0.001) and a similar 2.9-fold increase (p<0.001) versus CAHF-treated lean males. The other significant parameters that increased included (dp/dt)_max_ and LVDevP (Fig 3B, Table 2). The obese males exhibited a similar pattern as for the lean males and recovered similarly during the RAHF phase (Fig 3A and 3B, Table 3). We found no significant changes when comparing TMZ treatment for lean versus obese males (Table 4).

**Table 2:** The effects of TMZ treatment on lean male hearts subjected to AHF (n = 6).

**Treatment group**	**Heart rate (%)**	**Sig.**	**LVDevP (%)**	**Sig.**
Control	210.13 ± 127.11	ns	1259.06 ± 485.90	** ***
TMZ at CAHF	28.26 ± 1.48		2204.29 ± 355.33	
TMZ at RAHF	29.74 ± 2.23		6025.70 ± 654.65	
**Treatment group**	**LVEDP (%)**	**Sig.**	**LVESP (%)**	**Sig.**
Control	201.85 ± 36.57	ns	211.06 ± 43.49	ns
TMZ at CAHF	160.23 ± 9.38		170.36 ± 11.07	
TMZ at RAHF	158.26 ± 18.72		171.80 ± 24.07	

Data represent the last ten minutes of the RAHF phase and expressed as a percentage of CAHF. Significance is expressed as **p<0.01; ***p<0.001 vs. TMZ (at RAHF). LVDevP: left ventricular developed pressure; LVEDP: left ventricular end diastolic pressure; LVESP: left ventricular end systolic pressure; CAHF: critical acute heart failure; RAHF: recovery acute heart failure; Sig.: significance; ns: not significant.

**Table 3:** The effects of TMZ treatment on obese male hearts subjected to AHF (n = 6).

**Treatment group**	**Heart rate (%)**	**Sig.**	**LVDevP (%)**	**Sig.**
Control	31.75 ± 2.85	ns	2411.10 ± 458.16	**
TMZ at CAHF	30.17 ± 2.62		1678.49 ± 470.27	
TMZ at RAHF	31.78 ± 2.38		4522.35 ± 742.07	
**Treatment group**	**LVEDP (%)**	**Sig.**	**LVESP (%)**	**Sig.**
Control	142.48 ± 10.43	ns	147.98 ± 11.77	ns
TMZ at CAHF	158.98 ± 15.10		164.74 ± 16.48	
TMZ at RAHF	141.43 ± 9.98		147.96 ± 12.18	

Data represent the last ten minutes of the RAHF phase and expressed as a percentage of CAHF. Significance is expressed as **p<0.01 vs. TMZ at RAHF. LVDevP: left ventricular developed pressure; LVEDP: left ventricular end diastolic pressure; LVESP: left ventricular end systolic pressure; CAHF: critical acute heart failure; RAHF: recovery acute heart failure; Sig.: significance; ns: not significant.

**Table 4:** Comparison of TMZ effects on hearts subjected to AHF—Isolated from lean and obese males (n = 6).

	**Control**					
	**Lean**			**Obese**		**Sig.**
Heart rate (%)	210.13 ± 127.11			31.75 ± 2.85		ns
LVDevP (%)	1259.06 ± 485.90			2411.10 ± 458.16		ns
LVEDP (%)	201.85 ± 36.57			142.48 ± 10.43		ns
LVESP (%)	211.06 ± 43.49			147.98 ± 11.77		ns
	**TMZ at CAHF**			**TMZ at RAHF**		
	**Lean**	**Obese**	**Sig.**	**Lean**	**Obese**	**Sig.**
Heart rate (%)	28.26 ± 1.48	30.17 ± 2.62	ns	29.74 ± 2.23	31.78 ± 2.38	ns
LVDevP (%)	2204.29 ± 355.33	1678.49 ± 470.27	ns	6025.70 ± 654.65	4522.35 ± 742.07	ns
LVEDP (%)	160.23 ± 9.38	158.98 ± 15.10	ns	158.26 ± 18.72	141.43 ± 9.98	ns
LVESP (%)	170.36 ± 11.07	164.74 ± 16.48	ns	171.80 ± 24.07	147.96 ± 12.18	ns

Data represent the last ten minutes of the RAHF phase and expressed as a percentage of CAHF. LVDevP: left ventricular developed pressure; LVEDP: left ventricular end diastolic pressure; LVESP: left ventricular end systolic pressure; CAHF: critical acute heart failure; RAHF: recovery acute heart failure; Sig.: significance; ns: not significant.

### TMZ treatment of female hearts subjected to AHF

Females were divided into the two main stages of the estrus cycle, namely the follicular and luteal phase. However, obese females experienced stasis in the luteal phase. Thus we initially focused on differences between the phases of the lean animals followed by a comparison of lean and obese females (luteal phases).

#### TMZ treatment of lean female (follicular vs. luteal) hearts subjected to AHF

**Fig 4:**
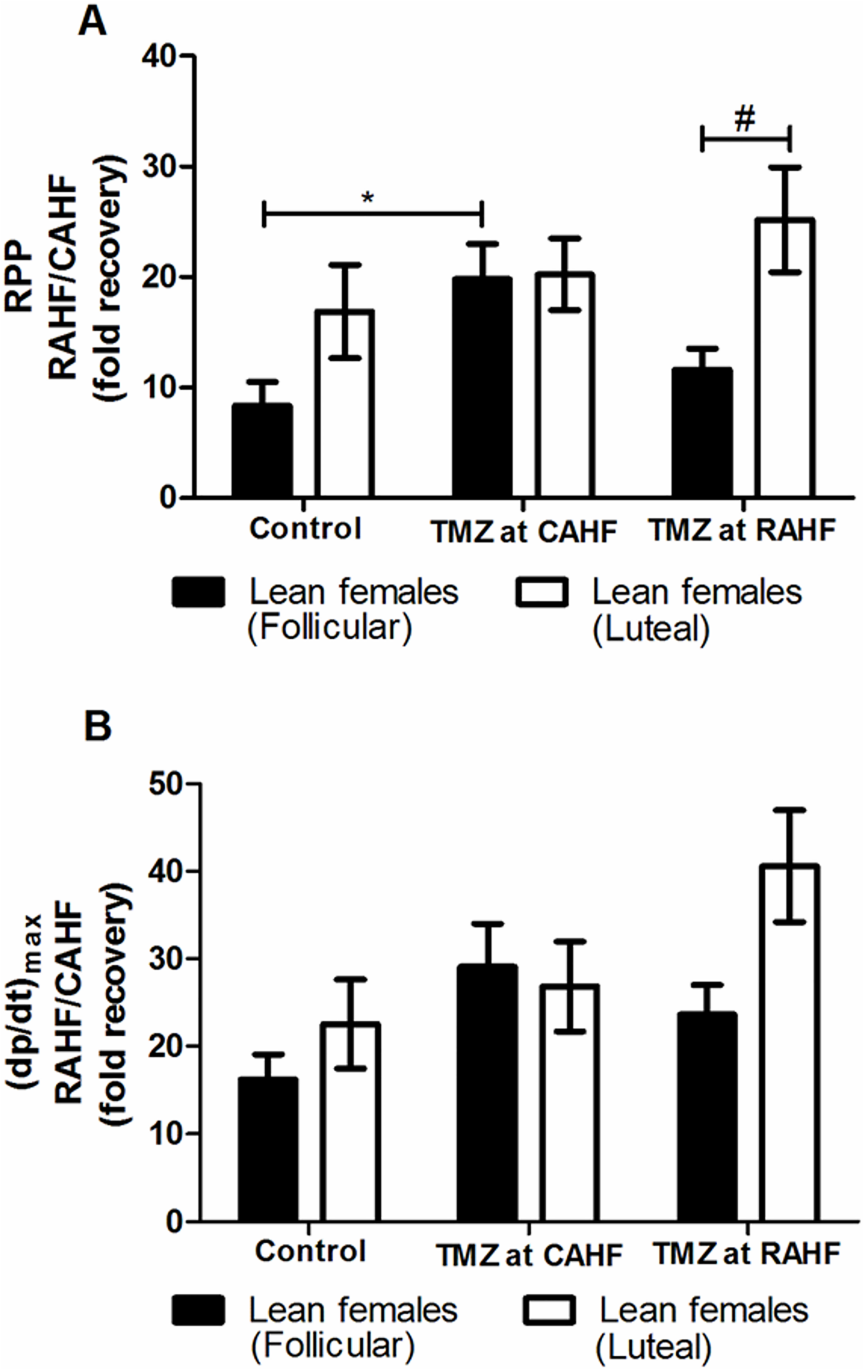
Effect of TMZ treatment on RPP and (dp/dt)_max_ of lean females (follicular and luteal phase). (A) RPP (B) (dp/dt)_max_ (n = 6). Data represent the last ten minutes of the RAHF phase and expressed as a fold recovery from the previous CAHF phase. Significance is expressed as *p<0.05 vs. control lean follicular females; and #p<0.05 vs. TMZ at RAHF (lean follicular females). RPP: rate pressure product; (dp/dt)_max_: index of myocardial contraction velocity; CAHF: critical acute heart failure; RAHF: recovery acute heart failure.

Mouse hearts from lean females (follicular) that received TMZ treatment during the CAHF phase displayed a robust increase in RPP (p<0.05) and LVDevP (p<0.05) compared to control females when function was assessed during the recovery phase (Fig 4A and 4B, Table 5). When TMZ treatment was initiated during the RAHF phase then this effect was blunted compared to initiation of treatment during the CAHF phase. Thus TMZ treatment of lean luteal females during the RAHF phase did not result in any significant changes (Fig 4A and 4B, Table 6). In addition, there were limited differences between lean luteal and follicular females except for TMZ treatment initiated in the RAHF phase where lean luteal females displayed a much higher RPP (p<0.05) (Fig 4A).

**Table 5:** Effect of TMZ treatment during the RAHF phase (lean follicular females) (n = 6).

**Treatment group**	**Heart rate (%)**	**Sig.**	**LVDevP (%)**	**Sig.**
Control	30.21 ± 4.14	ns	2875.81 ± 725.74	*
TMZ at CAHF	27.94 ± 3.51		8049.63 ± 1961.21	
TMZ at RAHF	23.93 ± 2.00		4918.75 ± 762.73	
**Treatment group**	**LVEDP (%)**	**Sig.**	**LVESP(%)**	**Sig.**
Control	140.87 ± 9.40	ns	149.10 ± 10.88	ns
TMZ at CAHF	139.35 ± 24.49		152.26 ± 30.49	
TMZ at RAHF	137.35 ± 3.71		145.29 ± 4.73	

Data represent the last ten minutes of the RAHF phase and expressed as a percentage of CAHF. Significance is expressed as *p<0.05 vs. control. LVDevP: left ventricular developed pressure; LVEDP: left ventricular end diastolic pressure; LVESP: left ventricular end systolic pressure; CAHF: critical acute heart failure; RAHF: recovery acute heart failure; Sig.: significance; ns: not significant.

**Table 6:** Effect of TMZ treatment during the RAHF phase (lean luteal females) (n = 6).

**Treatment group**	**Heart rate (%)**	**Sig.**	**LVDevP (%)**	**Sig.**
Control	33.55 ± 4.86	ns	6116.62 ± 1675.88	ns
TMZ at CAHF	34.18 ± 5.60		6202.00 ± 1064.64	
TMZ at RAHF	27.31 ± 1.41		9475.80 ± 1807.90	
**Treatment group**	**LVEDP (%)**	**Sig.**	**LVESP (%)**	**Sig.**
Control	179.64 ± 10.82	ns	199.59 ± 13.44	ns
TMZ at CAHF	176.24 ± 43.51		194.08 ± 51.29	
TMZ at RAHF	134.06 ± 4.27		147.00 ± 6.88	

Data represent the last ten minutes of the RAHF phase and is expressed as a percentage of CAHF. LVDevP: left ventricular developed pressure; LVEDP: left ventricular end diastolic pressure; LVESP: left ventricular end systolic pressure; CAHF: critical acute heart failure; RAHF: recovery acute heart failure; Sig.: significance; ns: not significant.

#### TMZ treatment of lean versus obese female (luteal) hearts subjected to AHF

**Fig 5:**
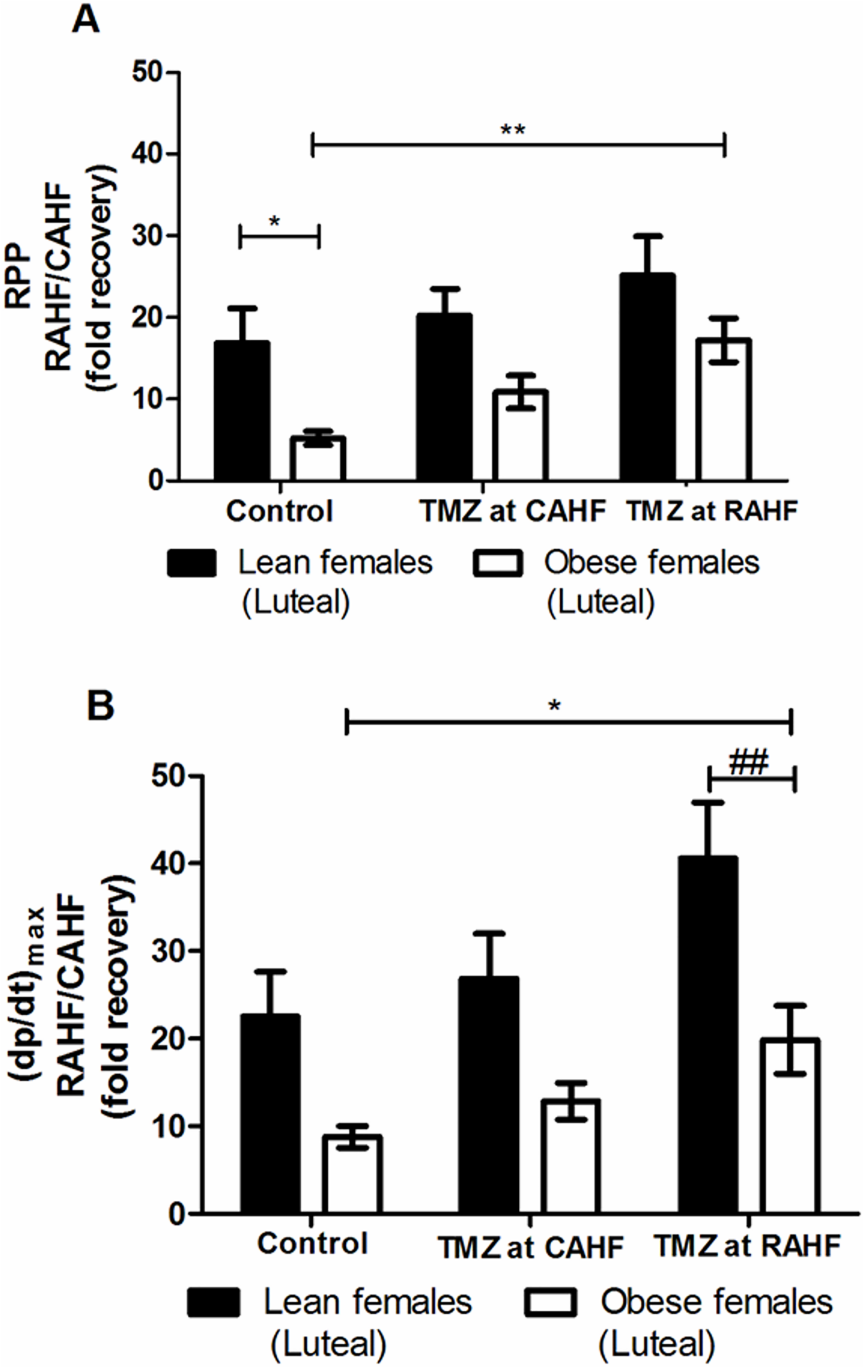
Effect TMZ treatment on RPP and (dp/dt)_max_ (lean vs. obese females). (A) RPP (B) (dp/dt)_max_ (n = 6). Data represent the last ten minutes of the RAHF phase and expressed as a fold recovery from the previous CAHF phase. Significance is expressed as *p<0.05; **p<0.01 vs. control obese females. RPP: rate pressure product; (dp/dt)_max_: index of myocardial contraction velocity; CAHF: critical acute heart failure; RAHF: recovery acute heart failure.

The control obese females showed a lower recovery after AHF compared to lean counterparts (Fig 5A and 5B). No significant differences were found between lean follicular and luteal females treated with TMZ (Table 7). However, obese females with TMZ treatment (initiated at RAHF) responded favorably with a 3.3-fold increase in RPP (p<0.01), a 2.3-fold increase in (dp/dt)_max_ (p<0.05) and a significant increase in LV developed pressure compared to control obese females (Fig 5A and 5B, Table 8). When comparing lean and obese luteal females during the RAHF phase it is evident that lean females consistently displayed a superior improvement in heart function. For example, the RPP is 3.2-fold (p<0.05) higher versus obese females while lean females that received TMZ treatment during the RAHF phase displayed a higher (dp/dt)_max_ (p<0.01) compared to obese females (Fig 5A and 5B, Table 9).

**Table 7:** Comparison of TMZ effects on hearts subjected to AHF—Lean follicular vs. luteal females (n = 6).

	**Control**					
**Parameters**	**Follicular**			**Luteal**		**Sig.**
Heart rate (%)	30.21 ± 4.14			33.55 ± 4.86		ns
LVDevP (%)	2875.81 ± 725.74			6116.62 ± 1675.88		ns
LVEDP (%)	140.87 ± 9.40			179.64 ± 10.82		ns
LVESP (%)	149.10 ± 10.88			199.59 ± 13.44		ns
	**TMZ at CAHF**			**TMZ at RAHF**		
	**Follicular**	**Luteal**	**Sig.**	**Follicular**	**Luteal**	**Sig.**
Heart rate (%)	27.94 ± 3.51	34.18 ± 5.60	ns	23.93 ± 2.00	27.31 ± 1.41	ns
LVDevP (%)	8049.63 ± 1961.21	6202.00 ± 1064.64	ns	4918.75 ± 762.73	9475.80 ± 1807.90	ns
LVEDP (%)	139.35 ± 24.49	176.24 ± 43.51	ns	137.35 ± 3.71	134.06 ± 4.27	ns
LVESP (%)	152.26 ± 30.49	194.08 ± 51.29	ns	145.29 ± 4.73	147.00 ± 6.88	ns

Data represent the last ten minutes of the RAHF phase and expressed as a percentage of CAHF. LVDevP: left ventricular developed pressure; LVEDP: left ventricular end diastolic pressure; LVESP: left ventricular end systolic pressure; CAHF: critical acute heart failure; RAHF: recovery acute heart failure; Sig.: significance; ns: not significant.

**Table 8:** Effect of TMZ treatment on hearts subjected to AHF—Obese luteal females (n = 6).

**Treatment group**	**Heart rate (%)**	**Sig.**	**LVDevP (%)**	**Sig.**
Control	38.49 ± 10.56	ns	1725.24 ± 397.04	**
TMZ at CAHF	36.99 ± 3.84		3199.25 ± 743.72	
TMZ at RAHF	32.55 ± 2.48		5400.37 ± 887.21	
**Treatment group**	**LVEDP (%)**	**Sig.**	**LVESP (%)**	**Sig.**
Control	574.86 ± 308.07	ns	521.49 ± 240.36	ns
TMZ at CAHF	140.93 ± 11.62		145.23 ± 12.91	
TMZ at RAHF	126.83 ± 7.53		132.12 ± 9.01	

Data represent the last ten minutes of the RAHF phase and expressed as a percentage of CAHF. Significance is expressed as **p<0.01 versus control. LVDevP: left ventricular developed pressure; LVEDP: left ventricular end diastolic pressure; LVESP: left ventricular end systolic pressure; CAHF: critical acute heart failure; RAHF: recovery acute heart failure; Sig.: significance; ns: not significant.

**Table 9:** Comparison of TMZ effects on hearts subjected to AHF—Lean vs. obese females (luteal) (n = 6).

	**Control**					
**Parameters**	**Lean**			**Obese**		**Sig.**
Heart rate (%)	33.55 ± 4.86			38.49 ± 10.56		ns
LVDevP (%)	6116.62 ± 1675.88			1725.24 ± 397.04		*
LVEDP (%)	179.64 ± 10.82			574.86 ±308.07		ns
LVESP (%)	199.59 ± 13.44			521.49 ± 240.36		ns
	**TMZ at CAHF**			**TMZ at RAHF**		
	**Lean**	**Obese**	**Sig.**	**Lean**	**Obese**	**Sig.**
Heart rate (%)	34.18 ± 5.60	36.99 ± 3.84	ns	27.31 ± 1.41	32.55 ± 2.48	ns
LVDevP (%)	6202.00 ± 1064.64	3199.25 ± 743.72	ns	9475.80 ± 1807.90	5400.37 ± 887.21	ns
LVEDP (%)	176.24 ± 43.51	140.93 ± 11.62	ns	134.06 ± 4.27	126.83 ± 7.53	ns
LVESP (%)	194.08 ± 51.29	145.23 ± 12.91	ns	147.00 ± 6.88	132.12 ± 9.01	ns

Data represent the last ten minutes of the RAHF phase and expressed as a percentage of CAHF. Significance is expressed as *p<0.05 vs. lean females. LVDevP: left ventricular developed pressure; LVEDP: left ventricular end diastolic pressure; LVESP: left ventricular end systolic pressure; CAHF: critical acute heart failure; RAHF: recovery acute heart failure; Sig.: significance; ns: not significant.

### Acute heart failure: Males vs. females

#### Lean groups

**Fig 6:**
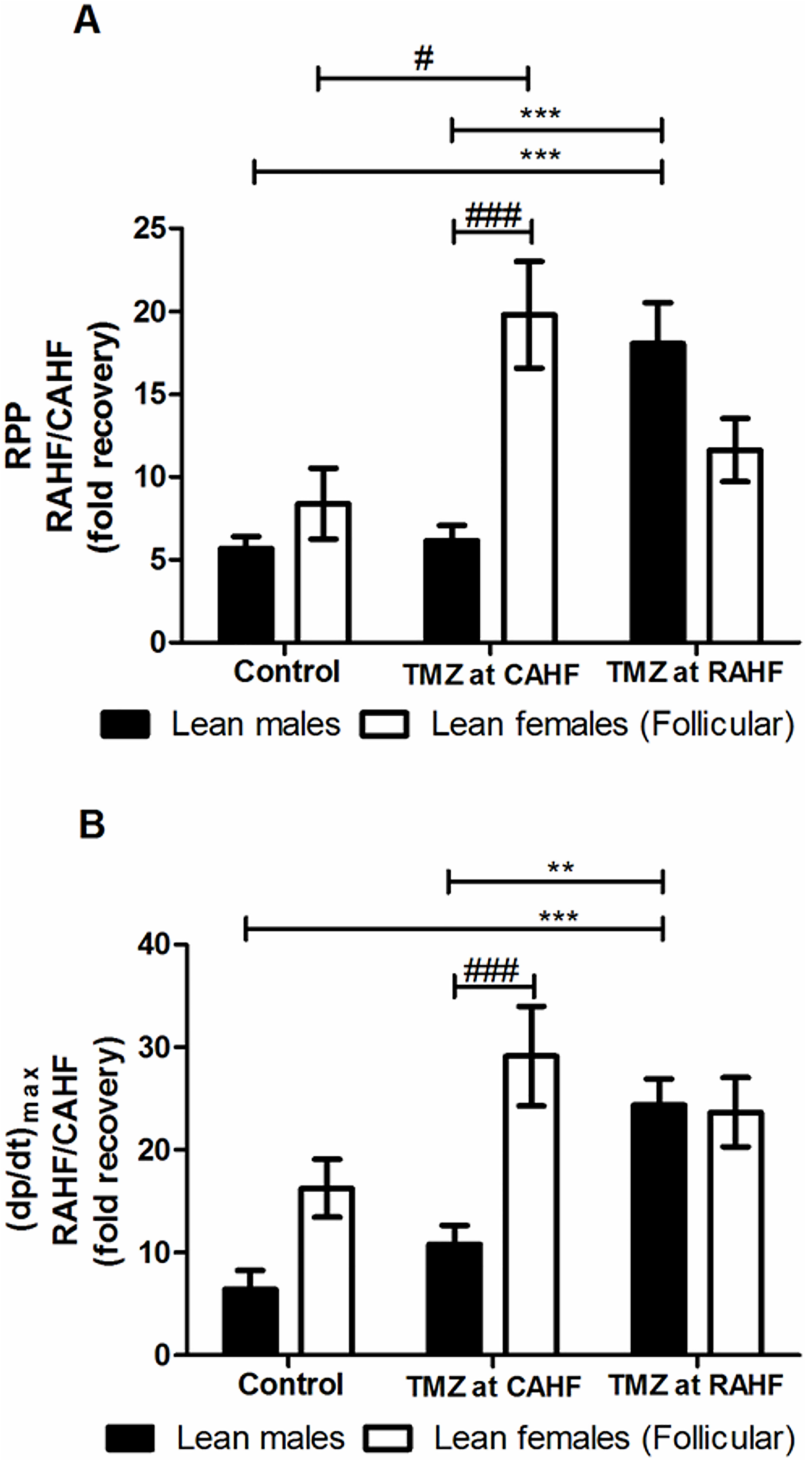
Effect of TMZ treatment on RPP and (dp/dt)_max_ of lean males vs. lean females (follicular). (A) RPP (B) (dp/dt)_max_ (n = 6). Data represent the last ten minutes of the RAHF phase and expressed as a fold recovery from the previous CAHF phase. Significance is expressed as **p<0.01; ***p<0.001 vs. TMZ-treated lean males (initiated during RAHF); and #p<0.05; ###p<0.001 vs. TMZ-treated lean males (initiated during CAHF). RPP: rate pressure product; (dp/dt)_max_: index of myocardial contraction velocity; CAHF: critical acute heart failure; RAHF: recovery acute heart failure.

We initially compared lean males with lean females (follicular) during the recovery phase (RAHF)–without any treatments. Here control lean males showed a 5.6- and 6.4-fold increase in RPP and (dp/dt)_max_, respectively, from the previous CAHF phase (Fig 6A and 6B). The lean control follicular females also displayed improved function and to a greater extent than their male counterparts, although such changes were not statistically significant. For the TMZ treatment regimens initiated during the CAHF phase, we found that females exhibited a much higher RPP (p<0.001), (dp/dt)_max_ (p<0.001) and LVDevP (p<0.001) versus lean males (Fig 6A and 6B, Table 10). For the RAHF-treated animals, there were no significant sex-based differences where only males benefited from TMZ treatment.

**Table 10:** Comparison of TMZ effects on hearts subjected to AHF—Lean males vs. lean females (follicular) (n = 6).

	**Control**					
**Parameters**	**Males**			**Females**		**Sig.**
Heart rate (%)	210.13 ± 127.11			30.21 ± 4.14		ns
LVDevP (%)	1259.06 ± 485.90			2875.81 ± 725.74		ns
LVEDP (%)	201.85 ±36.57			140.87 ± 9.40		ns
LVESP (%)	211.06 ± 43.49			149.10 ± 10.88		ns
	**TMZ at CAHF**			**TMZ at RAHF**		
	**Males**	**Females**	**Sig.**	**Males**	**Females**	**Sig.**
Heart rate (%)	28.26 ± 1.48	27.94 ± 3.51	ns	29.74 ± 2.23	23.93 ± 2.00	ns
LVDevP (%)	2204.29 ± 355.33	8049.63 ± 1961.21	***	6025.70 ± 654.65	4918.75 ± 762.73	ns
LVEDP (%)	160.23 ± 9.38	139.35 ± 24.49	ns	158.26 ± 18.72	137.35 ± 3.71	ns
LVESP (%)	170.36 ± 11.07	152.26 ± 30.49	ns	171.80 ± 24.07	145.29 ± 4.73	ns

Data represent the last ten minutes of the RAHF phase and is expressed as a percentage of CAHF. Significance is expressed as ***p<0.001 vs. lean males. LVDevP: left ventricular developed pressure; LVEDP: left ventricular end diastolic pressure; LVESP: left ventricular end systolic pressure; CAHF: critical acute heart failure; RAHF: recovery acute heart failure; Sig.: significance; ns: not significant.

**Fig 7:**
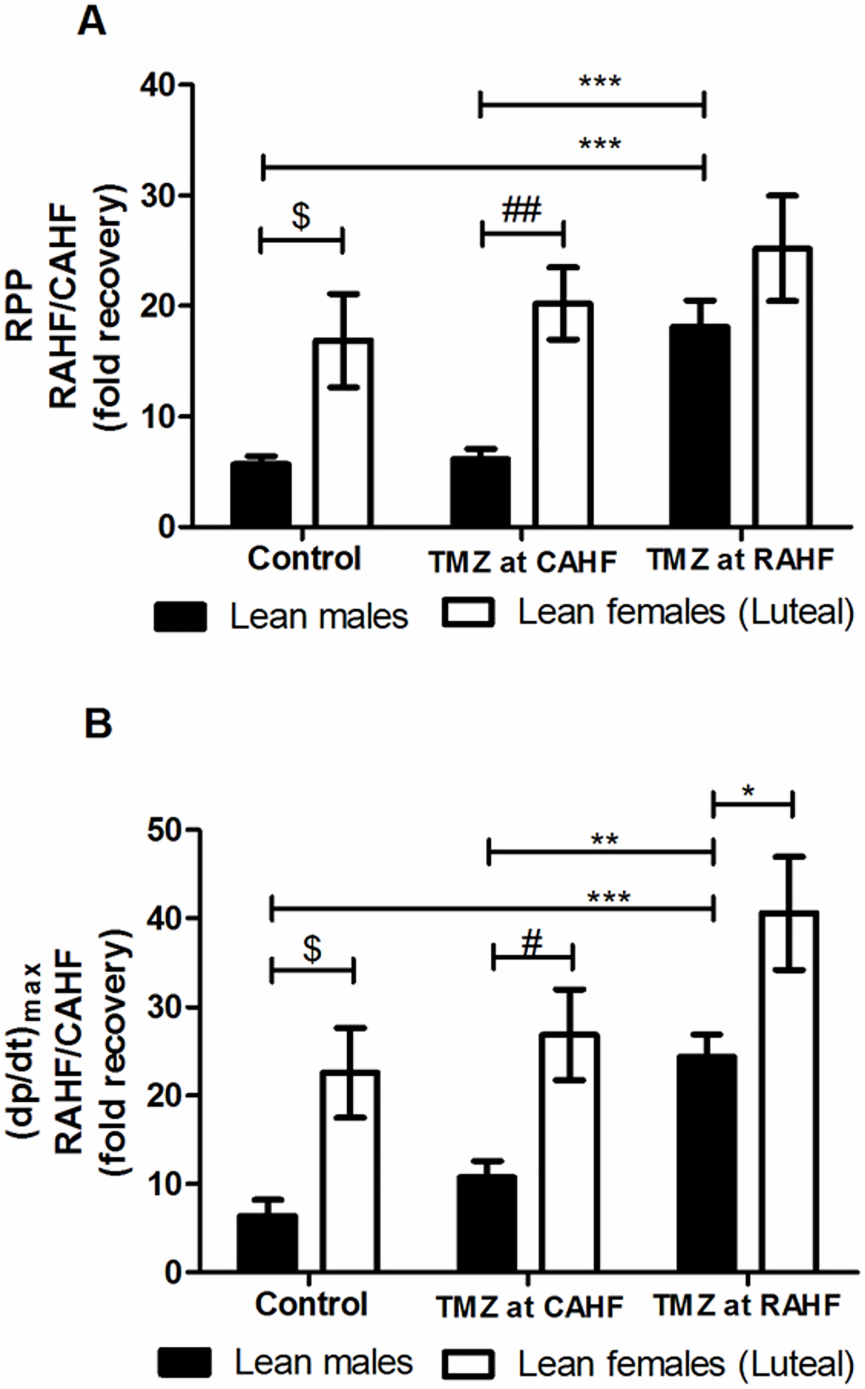
Effect of TMZ treatment on RPP and (dp/dt)_max_ of lean males vs. lean females (luteal). (A) RPP (B) (dp/dt)_max_ (n = 6). Data represent the last ten minutes of the RAHF phase and expressed as a fold recovery from the previous CAHF phase. Significance is expressed as *p<0.05; **p<0.01; ***p<0.001 vs. TMZ at RAHF lean males, #p<0.05; ##p<0.01 vs. TMZ at CAHF lean males; and $<0.05 vs. control lean males. RPP: rate pressure product; (dp/dt)_max_: index of myocardial contraction velocity; CAHF: critical acute heart failure; RAHF: recovery acute heart failure.

We next compared lean males with lean females (luteal) and as before, control males exhibited increased RPP and (dp/dt)_max_ from the previous CAHF phase (Fig 7A and 7B). The control females (luteal) showed a robust increase for both parameters that was significantly higher versus control lean males (Fig 7A and 7B, Table 11). However, this increase was not significantly different when compared to control follicular females (previously discussed). As for follicular females, luteal females treated with TMZ during the CAHF phase displayed a higher RPP (p<0.01) (dp/dt)_max_ (p<0.05) and LVDevP (p<0.05) compared to lean males. For TMZ initiated during the RAHF phase, lean males showed significant improvement vs. controls although RAHF-treated females still exhibited increased (dp/dt)_max_ (p<0.05) versus lean males (Fig 7B, Table 11).

**Table 11:** Comparison of TMZ effects on hearts subjected to AHF—Lean males vs. lean females (luteal) (n = 6).

	**Control**					
**Parameters**	**Males**			**Females**		**Sig.**
Heart rate (%)	210.13 ± 127.11			33.55 ± 4.86		ns
LVDevP (%)	1259.06 ± 485.90			6116.62 ± 1675.88		*
LVEDP (%)	201.85 ± 36.57			179.64 ± 10.82		ns
LVESP (%)	211.06 ± 43.49			199.59 ± 13.44		ns
	**TMZ at CAHF**			**TMZ at RAHF**		
	**Males**	**Females**	**Sig.**	**Males**	**Females**	**Sig.**
Heart rate (%)	28.26 ± 1.48	34.18 ± 5.60	ns	29.74 ± 2.23	27.31 ± 1.41	ns
LVDevP (%)	2204.29 ± 355.33	6202.00 ± 1064.64	ns	6025.70 ± 654.65	9475.80 ± 1807.90	ns
LVEDP (%)	160.23 ± 9.38	176.24 ± 43.51	ns	158.26 ± 18.72	134.06 ± 4.27	ns
LVESP (%)	170.36 ± 11.07	194.08 ± 51.29	ns	171.80 ± 24.07	147.00 ± 6.88	ns

Data represent the last ten minutes of the RAHF phase and expressed as a percentage of CAHF. Significance is expressed as *p<0.05. LVDevP: left ventricular developed pressure; LVEDP: left ventricular end diastolic pressure; LVESP: left ventricular end systolic pressure; CAHF: critical acute heart failure; RAHF: recovery acute heart failure; Sig.: significance; ns: not significant.

#### Obese groups

**Fig 8:**
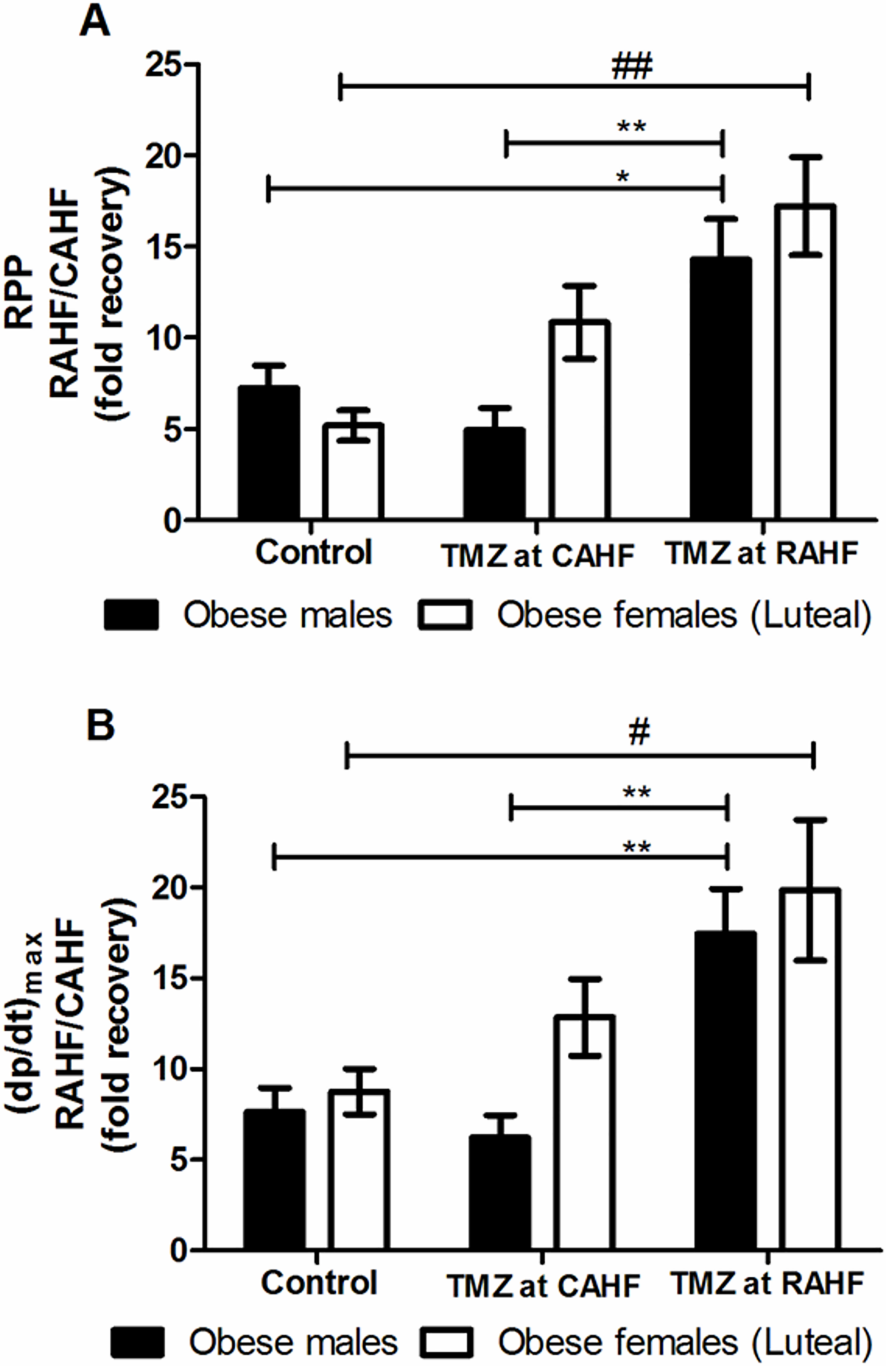
Effect of TMZ treatment on RPP and (dp/dt)_max_ of obese males and females (luteal). (A) RPP (B) (dp/dt)_max_ (n = 6). Data represent the last ten minutes of the RAHF phase and expressed as a fold recovery from the previous CAHF phase. Significance is expressed as *p<0.05, **p<0.01 vs. TMZ at RAHF for obese males; and #p<0.05, ##p<0.01 vs. TMZ at RAHF for obese females. RPP: rate pressure product; (dp/dt)_max_: index of myocardial contraction velocity; CAHF: critical acute heart failure; RAHF: recovery acute heart failure.

Obese females (luteal) treated with TMZ during CAHF did not display significant functional improvements as with the lean females (Fig 8A and 8B, Table 12). When treatment was initiated during the RAHF phase the females showed a significant improvement but to a similar degree when compared to obese males.

**Table 12:** Comparison of TMZ effects on hearts subjected to AHF–obese males vs. obese females (luteal) (n = 6).

	**Control**					
**Parameters**	**Males**			**Females**		**Sig.**
Heart rate (%)	31.75 ± 2.85			38.49 ± 10.56		ns
LVDevP (%)	2411.10 ± 458.16			1725.24 ± 397.04		ns
LVEDP (%)	142.48 ± 10.43			574.86 ± 308.07		ns
LVESP (%)	147.98 ± 11.77			521.49 ± 240.36		*
	**TMZ at CAHF**			**TMZ at RAHF**		
	**Males**	**Females**	**Sig.**	**Males**	**Females**	**Sig.**
Heart rate (%)	30.17 ± 2.62	36.99 ± 3.84	ns	31.78 ± 2.38	32.55 ± 2.48	ns
LVDevP (%)	1678.49 ± 470.27	3199.25 ± 743.72	ns	4522.35 ± 742.07	5400.37 ± 887.21	ns
LVEDP (%)	158.98 ± 15.10	140.93 ± 11.62	ns	141.43 ± 9.98	126.83 ± 7.53	ns
LVESP (%)	164.74 ± 16.48	145.23 ± 12.91	ns	147.96 ± 12.18	132.12 ± 9.01	ns

Data represent the last ten minutes of the RAHF phase and expressed as a percentage of CAHF. Significance is expressed as *p<0.05 vs. obese males. RPP: rate pressure product; (dp/dt)_max_: index of myocardial contraction velocity; LVDevP: left ventricular developed pressure; LVEDP: left ventricular end diastolic pressure; LVESP: left ventricular end systolic pressure; CAHF: critical acute heart failure; RAHF: recovery acute heart failure; Sig.: significance; ns: not significant.

## Discussion

We hypothesized that TMZ (partial FAO inhibitor) offers cardioprotection during AHF under normal and obese-related diabetic conditions and that this may be influenced in a sex-dependent manner. The main findings of this study are: (1) our perfusion system resulted in significant decreases in both functional and pressure parameters during the CAHF phase, reflecting *de novo* AHF. Moreover, our model reflected partial recovery in the RAHF phase with modest increases in functional and pressure parameters although this was still significantly lower than baseline values; (2) lean (*db*/+) females showed normal estrous cycle phases namely proestrus, estrus (follicular phase), metestrus and diestrus (luteal phase) while obese females (*db*/*db*) did not undergo the follicular phases (proestrus and estrus); and (3) TMZ treatment resulted in negligible changes to functional parameters during the CAHF phase, although significant results were obtained in terms of recovery function during the RAHF phase. Lean and obese males responded equally well to TMZ treatment. However, lean females (follicular and luteal phases) responded in distinct fashion, while obesity influenced females’ response to TMZ administration. Due to the unique response pattern of the different estrous phases, sex differences were present only in lean groups.

To establish an *ex vivo* mouse AHF model we began by modifying a previous experimental system of acutely underperfused rat hearts where AHF was simulated by decreasing coronary perfusion pressure [44–46]. This model is representative of hypotensive AHF patients who present with hypoperfusion, cardiogenic shock and low blood pressure [15, 20, 49]. Lower pressure upon admission is inversely correlated with in-hospital mortality [50]. In agreement with the original model, *de novo* AHF is induced in the isolated heart by decreasing the perfusion pressure from 100 cm to 20 cmH_2_O and by altering metabolic substrates in the perfusate in order to 1) simulate circulating metabolite conditions of HF patients and 2) promote the upregulation of FA utilization during heart failure. We therefore introduced elevated FA (1.2 mM/L palmitic acid conjugated to 3% BSA) [51, 52] and decreased the glucose concentration from 10 to 2.5 mM/L [53]. This perfusion system resulted in profound decreases in both functional and pressure parameters during the CAHF phase reflecting *de novo* AHF (Fig 2A and 2B and Table 1). The recovery phase was similarly manipulated by restoring the perfusion pressure back to 100 cmH_2_O, restoring glucose to baseline levels (10 mM/L) and by maintaining the high FA content. The model reflected partial recovery during the RAHF phase (Fig 2A and 2B and Table 1) with modest increases in functional and pressure parameters that were still significantly lower than baseline values.

In order to evaluate whether the estrous cycle may impact on TMZ efficacy during an AHF event, the different phases of the estrous cycle were initially investigated by vaginal cytology [40, 41]. The short estrous cycle (lasting 4–5 days) makes the mouse an ideal model to investigate influences of the reproductive cycle. After exposing the females to male pheromones to ensure regular cycling [42, 43], lean (*db*/+) females showed normal estrous cycle phases (Fig 1) namely proestrus, estrus (follicular phase), metestrus and diestrus (luteal phase). However, obese females (*db*/*db*) did not undergo the follicular phases (proestrus and estrus), but rather displayed metestrus and diestrus acyclicity, a phenomenon common in this transgenic mouse strain [54–56].

Our results showed that lean and obese males recovered in a similar manner at both treatment time points, i.e. TMZ treatment during the CAHF and RAHF phases, respectively (Fig 3A and 3B). This finding is in accordance to a study performed in streptozotocin-induced diabetic rats, where they also found no hemodynamic (peak systolic pressure, end diastolic pressure and dp/dt_max_) differences between non-diabetic and diabetic groups [57]. Groups receiving TMZ during the CAHF phase showed no statistical differences in heart function during their recovery as was expected (Table 13); TMZ treatment during the CAHF phase would partially inhibit 3-KAT and consequently increase glucose utilization [25, 58, 59]. However, during the CAHF phase glucose availability is exceptionally low and TMZ treatment shifts metabolism away from the only plentiful metabolite available to the heart. At this point there may be a neutralizing or even a possible counter-effect of TMZ treatment during the CAHF phase [60, 61].

**Table 13:** AHF *ex vivo* model–underlying features.

Baseline stabilization	CAHF	RAHF
***Simulated metabolism***		
Normal glucose	Low glucose	Normal glucose
No FAs	FAs	FAs
(Normal fuel)	(Push towards FAO)	(Choice in fuel)
***Induced functional parameters***		
Normal pressure	Low pressure	Normal pressure
Normal perfusion	Low perfusion	Normal perfusion
Paced	Unpaced	Paced
***Expected function under simulated conditions***		
Normal	Critically low	Increased function (recovery)
***Expected function with TMZ treatment***		
	Restore equilibrium back to glucose utilization	Favors the equilibrium towards glucose utilization

FAs: fatty acids; FAO: Fatty acid β-oxidation

Treatment during the RAHF phase, however, resulted in significant functional recovery (Tables 2 and 3). Metabolic therapies (e.g. TMZ) are extensive areas of research and have been successful for CVD treatment, with the latter attributed to the promotion of glucose utilization restoring homeostasis and alleviating the detrimental effect of increased FAO [26, 62, 63, 64, 65]. Liu *et al*. (2016) showed that TMZ administration decreased myocardial infarction in mice by significantly activating AMPK and ACC and by shifting the metabolism from FAO to GO [66]. TMZ also increased mechanical heart function in *db/db* mice by attenuating FAO-mediated lipotoxicity and oxidative stress, and also suppressing and preventing the development of diabetic cardiomyopathy [67,68]. This was confirmed in obese humans, where TMZ improved myocardial efficiency by decreasing FAO [69]. Our results also demonstrate that obesity does not influence TMZ efficacy for AHF treatment in male mice (Fig 3A and 3B and Table 4). However, some reported contrarian findings, i.e. failing hearts being more dependent on FFA availability. For example, studies on chronic failing hearts and cardiomyopathic heart failure showed that decreased FFA during HF negatively impacts on both myocardial efficiency and energy metabolism [60, 61]. Together these data show that the degree of FAO inhibition is crucial to ensure sufficient availability of fuel substrates to still allow for myocardial ATP generation and adequate contractile function. Here TMZ as a well-known partial FAO inhibitor seems to match these criteria quite well.

Lean females in the two different phases of the estrous cycle responded in unique ways (Fig 4A and 4B). Here lean follicular females recovered optimally when treated with TMZ during the CAHF phase, unlike when there was no significant recovery when the hearts were treated during the RAHF phase (Table 6). The lean luteal females were the only group to exhibit inherent superior functional recovery and were also the only group that was not significantly affected by TMZ treatment for any of the treatment time points (Table 6). This led us to conclude that this treatment difference may be due to the effect of sex hormones, i.e. the follicular phase (dominated by estrogen) and the luteal phase (characterized by progesterone) [40–42, 70]. The lean follicular females was the only group to show significant recovery when TMZ was administered during the CAHF phase. As previously discussed, the model employed shifts metabolism towards FAO during CAHF, but TMZ treatment would encourage less desirable utilization of glucose that is severely decreased (Table 13). We speculate that the beneficial effects of TMZ could be via estrogen increasing FAO and decreasing glucose utilization [36], thus allowing the myocardium to utilize the high FAs during this phase. In support, females seem to be better adapted to utilize FA and may also be protected against increased FA levels compared to males [35].

Our results revealed that the lean luteal females did not respond to TMZ treatment. Research in sex differences in pharmacokinetics and pharmacodynamics [71–72] demonstrated that increased sex hormones in women can alter hepatic enzyme activities leading to fluctuations in drug accumulation and/or elimination. Drug metabolism can be altered during the latter stages of the luteal phase as estrogen is a substrate for cytochrome P450, family 3, subfamily A (CYP3A) [72,73]. This may explain the lack of response to TMZ in our model as the biotransformation of TMZ is speculated to involve isoenzymes of CYP3A [74,75] The findings generated in this study also demonstrate that lean luteal females possess an inherent “coping mechanism” to better tolerate the AHF event, despite the lack of response to TMZ treatment. This could be attributed to the protective effects of progesterone on the cardiovascular system [76], e.g. it can assist in cardiac repair by enhancing fibroblast regeneration and differentiation [77]. Moreover, progesterone displays greater counter-regulatory responses in response to adrenergic stimulae [78], i.e. it can antagonize vasoconstriction [79], is associated with increased blood flow [80] and can decrease arrhythmias and the risk of sudden cardiac death [81]. We propose that any of these factors could influence the apparent innate protection observed in the lean luteal females. Intriguingly, this inherent cardioprotection is lost with obesity in luteal females across all the treatment groups (Fig 5A and 5B and Table 9). Here obese females exhibited the same treatment pattern as their lean counterparts, but at lower values (Fig 5A and 5B and Table 8). Furthermore, obese luteal females responded to TMZ treatment in the same manner as the males and benefitted only when it was added during the RAHF phase. We speculate that the treatment effect in females may be affected by the degree of obesity as obese female mice displayed a 20% increase in body weight compared to 10% for males [55]. In addition, specific sex differences in body fat content and protein binding can affect distribution of drugs [71, 82].

Our results revealed that sex differences in TMZ efficacy as a therapy for AHF is only present in lean groups (Figs 6 and 7 and Tables 10 and 11). There were no differences in functional recovery between obese males and females (Fig 8A and 8B and Table 12), showing that obesity is detrimental to both sexes. Sex differences are present between the lean males, and both follicular and luteal lean females. Sex differences in pharmacokinetics and pharmacodynamics is a common and prevailing phenomenon and elicited considerable interest [72–73]. Alterations can arise due to sex differences in drug absorption (gastric and hepatic enzyme activities, transporter proteins, and handling of drugs or metabolites), drug distribution (body fat composition and cardiac output), drug metabolism (cytochrome P450 group, hepatic and extra-hepatic metabolism) and drug elimination. We propose that the distinct sex differences found in the two estrus phases may be due to varying treatment patterns as a result of sex hormone differences.

Limitations to the current study include the lack of measurement of steroid levels and should ideally include suitable methods to evaluate estradiol and progesterone concentrations. Additionally, inclusion of an experimental group where TMZ is administered during the CAHF phase followed by a) a ‘‘wash out” period and b) a non-TMZ RAHF phase would have strengthened our findings. Based on our results showing that some females are resistant to TMZ treatment, additional evaluation of increased drug dosages in this instance may eventually trigger cardioprotection. Future *in vivo* studies should also evaluate acute TMZ administration to db/db mice and its effects on cardiac metabolism and heart function.

## Conclusions

We have successfully established a novel *ex vivo* mouse AHF model and these data reveal that TMZ elicits negligible effects on heart function during the CAHF phase, but that it results in significant recovery for both male and obese female hearts when administered during the RAHF phase. Our findings demonstrate that there were no differences between lean and obese male hearts, while lean females are at a clear functional recovery advantage over lean males. Here the luteal group displayed superior functional improvement and this is likely due to an innate coping mechanism that is lost with obesity. Thus TMZ emerges as a worthy therapeutic target to consider for AHF treatment in normal and obese-diabetic individuals (for both sexes), but only when administered during the recovery phase and not during the very acute stages.
